# Interleukin-1 Gene Polymorphisms in Chronic Gastritis Patients Infected with *Helicobacter pylori* as Risk Factors of Gastric Cancer Development

**DOI:** 10.1007/s00005-013-0245-y

**Published:** 2013-08-31

**Authors:** Andrzej Hnatyszyn, Karolina Wielgus, Marta Kaczmarek-Rys, Marzena Skrzypczak-Zielinska, Marlena Szalata, Joanna Mikolajczyk-Stecyna, Jerzy Stanczyk, Ireneusz Dziuba, Adam Mikstacki, Ryszard Slomski

**Affiliations:** 1 Department of Internal Medicine, District Hospital, Drawsko Pomorskie, Poland; 2 Independent Public Hospital, Health Care Centre, Nowa Sol, Poland; 3 Department of Biotechnology, Institute of Natural Fibres and Medicinal Plants, Poznan, Poland; 4 Institute of Human Genetics, Polish Academy of Sciences, Strzeszynska 32, 60-479 Poznan, Poland; 5 Department of Biochemistry and Biotechnology, Poznan University of Life Sciences, Poznan, Poland; 6 Department of Pathology, Regional Hospital, Gorzow Wlkp, Poland; 7 Department of Genetics and Pathology, Pomeranian Medical University, Szczecin, Poland; 8 Intensive Care Unit, Regional Hospital, Poznan, Poland

**Keywords:** *Helicobacter pylori*, Interleukin-1, Polymorphism, Chronic gastritis, Gastric cancer

## Abstract

Epidemiological investigations indicated association of the *Helicobacter pylori* infections with the occurrence of inflammatory conditions of the gastric mucosa and development of chronic gastritis and intestinal type of gastric cancer. *IL1A* and *IL1B* genes have been proposed as key factors in determining risk of gastritis and malignant transformation. The aim of this paper was to evaluate association of interleukin-1 gene polymorphisms with chronic gastritis, atrophy, intestinal metaplasia, dysplasia and intestinal type of gastric cancer in *H. pylori-*infected patients. Patients subjected to analysis represent group of 144 consecutive cases that suffered from dyspepsia with coexisting infection of *H. pylori* and chronic gastritis, chronic atrophic gastritis, intestinal metaplasia, dysplasia or gastric cancer. Molecular studies involved analysis of –889C>T polymorphism of *IL1A* gene and +3954C>T polymorphism of *IL1B* gene. Statistical analysis of association of polymorphism –889C>T of gene *IL1A* with changes in gastric mucosa showed lack of significance, whereas +3954C>T polymorphism of *IL1B* gene showed significant association. Frequency of allele T of +3954C>T polymorphism of *IL1B* gene was higher in group of patients with chronic gastritis, atrophy, intestinal metaplasia, dysplasia or intestinal type of gastric cancer (32.1 %) as compared with population group (23 %), χ^2^ = 4.61 and *p* = 0.03. This corresponds to odds ratio: 1.58, 95 % CI: 1.04–2.4. Our results indicate that +3954C>T polymorphism of *IL1B* gene increase susceptibility to inflammatory response of gastric mucosa *H. pylori*-infected patients and plays a significant role in the development of chronic gastritis, atrophy, intestinal metaplasia, dysplasia and the initiation of carcinogenesis.

## Introduction

The development of inflammatory conditions of the gastric mucosa is a complex and multi factorial process associated with balance disturbances between aggression and defence compounds. The diet, bile acids, hydrochloric acid as well as nonsteroidal anti-inflammatory drugs play a vital role in this process. A strong link is evident from epidemiological investigations between chronic inflammation and cancer (Bakwill and Mantovani 2001). The development of chronic gastritis is particularly strongly associated with the infection of *Helicobacter pylori* whose link with the spread of cancer has been confirmed beyond any doubt (IARC Working Group 1994; Kusić et al. 2003; Williams and Pounder 1999). A significant achievement leading to the recognition of mechanisms causing the disease was the isolation and culturing, in 1983 by Warren and Marshall, from the gastric mucosa of *Campylobacter pylori* bacteria. In their studies, they documented a link between the infection and gastritis (Warren and Marshall 1983). Active chronic gastric mucosa inflammation implicates, simultaneously, a pathogenic association with gastric ulcer and gastric cancer. Today, the significance of the *H. pylori* infection in the development of the stomach chronic inflammation and cancer is unquestionable (The EUROGAST study group 1993; Helicobacter and Cancer Collaborative Group 2001; Forman et al. 1991). In 1994, on the basis of many epidemiological experiments, the International Agency for Research on Cancer classified *H. pylori* as the first group carcinogen (IARC Working Group 1994). It is widely accepted that gastric carcinoma develops in many stages beginning from chronic inflammation, atrophic inflammation, intestinal metaplasia, dysplasia and, finally, into cancer. The above sequence of transformations in the gastric carcinoma carcinogenesis is commonly known as Correa cascade (Correa 1992). Smoking, diet, high salt consumption as well as other environmental factors should be treated as etiological causes contributing to the intensification of this process. However, infection of the *H. pylori* is an important triggering-off mechanism. Nevertheless, the development of the chronic inflammation of the gastric mucosa and, consequently, gastric carcinoma can also be affected by an individual, genetically preconditioned response to the infection. One of the consequences of *H. pylori* presence, like in case of other infectious agents, especially viruses, is change of methylation profile of infected tissue, which can lead to precancerous state (De Falco et al. 2011; Maekita et al. 2006).

Our recent analysis of *NOD2/CARD15* gene showed that the frequency of the T allele in 802C/T polymorphism was significantly higher (32.8 %) in the group of patients in comparison with the general population group (18.1 %), with relative risk of 1.8. In the patient group, the frequency of the CC genotype was 51.1 %, CT 32.1 % and TT 16.8 % (relative risk: 0.7, 1.1 and 4.2, respectively), while in the population group: 69.0, 25.7 and 5.3 % (relative risk: 1.0, 0.9 and 1.3, respectively) (Hnatyszyn et al. 2010).

Proinflammatory interleukin (IL)-1 induced by the *H. pylori* infection is representative of this process (Yamaoka et al. 1997). ILs belong to a diverse family of cytokines and represent specific cell signalling proteins which regulate the immune system of an organism. There are 37 interleukins identified in humans. The family of the IL-1 gene contains the three mutually inter-linked genes on the chromosome 2q: *IL1A*, *IL1B*, *IL1RN* encoding proinflammatory cytokines: IL-1α (IL1A), IL-1β (IL1B) as well as their receptor antagonist IL1RN (Dinarello 1996; El-Omar et al. 2000; Hurme et al. 1998; Nicklin et al. 1994). In the presence of *H. pylori*, the IL1B initiates and enhances the inflammatory response to the infection (Jung et al. 1997; Zambon et al. 2002). In addition, this cytokine strongly inhibits the secretion of the gastric acid; the inhibition is 100 times stronger in comparison with the proton pump inhibitors (Beales and Calam^1998^; Wallace et al. 1991) and 6,000 times stronger than the H2 receptor antagonists (Wolfe and Nompleggi 1992). This causes hypochlorhydria, decrease of the vitamin C level and increase of the gastrin concentration. In such conditions, nitrocompounds which play an important role in the intensification of the inflammatory process develop quite easily leading to the development of atrophic inflammation and increase in the occurrence of gastric carcinoma (El-Omar et al. 2000; Sobala et al. 1991; Zambon et al. 2002). Recently, attention has been focused on the existence of genetic polymorphism in humans and its significance for the predisposition towards the development of pathogenic states associated with *H. pylori* infection.

The IL1B gene (*IL1B*) is highly polymorphic. Transitions –511C/T and –31T/C were identified in the promoter region and transition +3954C/T in exon 5 (di Giovine et al. 1992; Pociot et al. 1992; Santtila et al. 1998). Synergistic interactions between carriers of the IL1B–511T and IL1RN*2 genotypes result in increased production of IL1B (Hurme et al. 1998; Hwang et al. 2002; Nishimura et al. 2002; Santtila et al. 1998). In their reports, El-Omar et al. (2000, 2001) demonstrated that the proinflammatory genotype (IL1B–511T, IL1B–31C and IL1RN*2) of the *IL1* gene occurs together with the increased risk of gastric carcinoma as well as its probable precursors of atrophic gastric inflammation and hypochlorhydria in the case of Polish and Scottish populations. This was further confirmed by other investigations on the Caucasian race carried out in Portugal (Figueiredo et al. 2002; Machado et al. 2001, 2003). Similar conclusions were drawn from experiments carried out on Japanese (Furuta et al. 2002) and Chinese (Yang et al. 2004) populations. However, there are also research reports which do not fully corroborate such correlations (Rad et al. 2004; Zeng et al. 2003). In another study, increased expression of the IL1B as well as intensification of the inflammatory reaction of the gastric mucosa was observed in carriers of the IL-1 proinflammatory polymorphism (IL1RN*2, IL1B–511T/–31C) (El-Omar et al. 2000, 2001). In addition, increased frequency of occurrence of intestinal metaplasia and gastric atrophic inflammation was also observed (Matsukura et al. 2003). Hwang et al. (2002) found that in Japanese patients infected with *H. pylori* who were carriers of the *IL1B*–*511T/T* gene polymorphism, alternatively carriers of the *IL1RN2* alleles, higher IL1B levels occurred in the gastric mucosa in comparison with non-carriers. On the other hand, Matsukura et al. (2003) reported ethnic differences in the impact of the *IL1B* gene polymorphism on the development of the atrophic inflammation of the gastric mucosa in Japanese, Chinese, Thai and Vietnamese populations. Gehmert et al. (2009) studied population from Peru, genotypically different from others studied and characterised by a high prevalence of *H. pylori* infection and gastric cancer. He conducted case–control study comparing 334 hospitalised patients with atrophic gastritis or gastric cancer with 158 nonatrophic gastritis patients (controls). Conditional logistic regression analysis revealed that an increased risk of atrophic gastritis (odds ratio [OR]: 5.60) and gastric cancer (OR: 2.36) was associated with the *IL1B*–*511C* allele.

This suggests that gene polymorphism of the host inflammatory response may affect the character and extent of the gastric mucosa associated with the *H. pylori* infection. The aim of our investigations was the analysis of the associations of cytokine proinflammatory genes: *IL1A* and *IL1B* with the occurrence of changes in the gastric mucosa. For this purpose, allele and genotype frequency of the *IL1A* gene polymorphism –889C>T and of the *IL1B* gene polymorphism +3954C>T was compared between general Polish population and patients with chronic gastritis, atrophy, intestinal metaplasia, dysplasia, intestinal type of gastric cancer with *H. pylori* infection.

## Materials and Methods

### Patients

Patients subjected to analysis represent group of 144 consecutive cases aged 11–87 collected in Western region of Poland. All patients suffered from dyspepsia with coexisting infection of *H. pylori* and chronic gastritis, chronic atrophic gastritis, intestinal metaplasia, dysplasia or gastric cancer. The investigations excluded patients taking, on a long-term basis, non-steroid anti-inflammatory drugs, drugs reducing gastric secretion, antibiotics and anti-coagulants during the previous 2 months as well as patients abusing alcohol and smoking tobacco, patients with serious somatic ailments (diseases of the liver, kidneys, cardiovascular and respiratory systems, etc.), patients after operations on bile ducts and gastric resections and active ulcer disease (gastric ulcer and duodenal ulcer). Authors selected control group of 13 patients without lesions and histological changes and without infection of *H. pylori* in gastric mucosa. Population group included 50 males and 50 females. Individuals from population group did not undergo endoscopy since at their examination and blood drawing they informed about lack of family history and they have not reported any symptoms from digestive tract. Characterisation of the examined groups is presented in Table 1. Population consisted of a group of healthy volunteers, from Laboratory of Molecular Genetics performing routine analysis for legal aspects. All persons have agreed with written consent to use their DNA for research. At the time of blood collection they showed no signs of any diseases and were negative for immunological test for *H. pylori* detection. Gastroscopy in the population group would be very helpful but was not performed due to the lack of acceptance of the invasive test for people without any symptoms of gastrointestinal disease by the Bioethic Commission.

**Table 1:** Demographic and clinical characteristics of the studied population. All patients were tested *Helicobacter pylori*-positive

Group of patients	Number of patients	Gender		Age (years)	*p* ^*^
		Females	Males		
Control group, without lesions and without infection of *H. pylori* in gastric mucosa	13	7 (54 %)	6 (46 %)	16–57 37.8 (±12.9)	0.9875
Chronic gastritis	40	20 (50 %)	20 (50 %)	16–75 35.6 (±14.6)	0.2565
Chronic gastritis with atrophy	36	19 (53 %)	17 (47 %)	11–76 41.0 (±18.5)	0.1752
Chronic gastritis with intestinal metaplasia	17	8 (47 %)	9 (53 %)	13–81 38.4 (±17.2)	0.9528
Chronic gastritis with dysplasia	21	12 (57 %)	9 (43 %)	17–87 52.5 (±18.7)	0.5267
Gastric cancer intestinal type	17	5 (29 %)	12 (71 %)	42–84 62.2 (±12.5)	0.4914
Population group	100	50 (50 %)	50 (50 %)	21–30 25.5 (±2.9)	0.0566

Females vs. males

### Gastroscopic Examinations

All endoscopic examinations of the stomach were performed in local anaesthesia (2 % Lignocainum) using videogastroscope (GiF Q 165 Olympus, Tokyo, Japan) by the same endoscopist. None of the patients was subjected to general anaesthesia. The macroscopic assessment of the endoscope image of the inflammatory changes of the gastric mucosa (oedema, hyperaemia, granulation of mucosa, presence of erosions and ulcers, hypertrophy or atrophy of gastric folds) was carried out in accordance with updated Sydney classification (Dixon et al. 1996; Misiewicz 1991). The following five tissue samples were taken in the course of the performed examinations: from the region of the pylorus, angle and corpus of the stomach from the larger and smaller curvature. Additional two biopsies from antrum and from corpus of the stomach were taken for *H. pylori* detection using rapid urease test (Institute of Food and Nutrition, Warsaw). Experiments were accepted by the Bioethic Commission of the Poznan University of Medical Sciences. All patients as well as parents and children had access to full information concerning the experiments and submitted their written consent for their performance.

### Histological Examinations

Histological assessment was performed by treatment of preparations with haematoxylin and eosin as described earlier (Hnatyszyn et al. 2010). All specimens were examined by the same histopathologist without clinical information. *H. pylori* infection was judged as positive by serum test or histological examination using Giemsa staining. The intensity of gastric mucosal inflammation, atrophy of glands, intestinal metaplasia and dysplasia were evaluated in each section according to updated Sydney classification (Misiewicz 1991; Dixon et al. 1996). The degree of mucosal inflammation, atrophy and intestinal metaplasia was classified using four grades as follows: 0–none; 1–mild; 2–moderate; and 3–severe. Gastric carcinoma was classified into intestinal and diffuse types according to Lauren’s criteria (Lauren 1965).

### Detection of the *H. pylori* Infection

The presence of the *H. pylori* bacteria was found using urease test (Institute of Food and Nutrition, Warsaw) which was read after 2 and 24 h (doubtful cases). Additionally, the presence of *H. pylori* bacteria was confirmed in the gastric mucosa by histological examinations employing the Warthin-Starry method modified by Giemsa according to updated Sydney system (Misiewicz 1991; Dixon et al. 1996). For all patients both tests were performed. The most essential results were achieved by histopathological examination, considering lower sensitivity and specificity of rapid urease test. Detection of *H. pylori* bacteria in population group was performed using immunological test (HELICO Test, Ani Biotech Oy, Finland). This test measures level of IgG antibodies against bacterium.

### Analysis of Polymorphism in *IL1A* and *IL1B* Genes

Molecular studies of association of –889C/T polymorphisms of *IL1A* and +3954C/T polymorphism of *IL1B* genes with the chronic gastritis and predisposition to cancer in *H. pylori*-infected patients were performed for patients with clinical diagnosis of different stages of chronic inflammation confirmed by gastroscopic and histological analyses. DNA was isolated in case of patients and control groups from paraffin blocks of gastric mucosal biopsies using Roche High Pure PCR template preparation kit and from peripheral blood using the guanidine isothiocyanate (GTC method).

Genotyping was performed using PCR–RFLP assay followed by electrophoresis in 6 % polyacrylamide gels (ALFExpress) or in 1.5 % agarose gels. In order to amplify 98 bp fragment encompassing –889C/T polymorphism of *IL1A* gene, the PCR reaction was performed in 20 μl of the reaction mixture containing 100 ng genomic DNA, 15 pmol of F and R primers each, 0.125 mM dNTP, 1.0 U Taq polymerase. Primer sequences were as follows: IL1A(–889C/T)F 5′-Cy5-GTTCTACCACCTGAACTAGGC-3′ and IL1A(–889C/T)R 5′-TTACATATGAGCCTTCC-ATG-3′. The 230-bp fragment encompassing +3954C/T polymorphism of *IL1B* gene was amplified using primers: IL1B(+3954C/T)F 5′-Cy5-GACTTTGACCGTATATGCTCAG-3′ and IL1B(+3954C/T)R 5′-ATGGACCAGACATCACCAAGC-3′. Reaction conditions: initial denaturation 95 °C, 5 min; 30 cycles: denaturation 92 °C, 30 s, primer annealing 55 °C, 45 s, synthesis 72 °C, 60 s and final synthesis 72 °C, 5 min. DNA fragments obtained after hydrolysis with restriction enzymes *Nco*I (37 °C, 6 h) and *Taq*I (65 °C, 6 h) were of the following sizes: allele *Nco*I(−) of *IL1A* gene 98 bp and allele *Nco*I(+) 82 and 16 bp, whereas allele *Taq*I(−) of *IL1B* gene 230 bp, allele *Taq*I(+) 126 and 104 bp.

### Statistical Analysis

If it is not indicated otherwise, the χ^2^ Pearson’s test was utilized for the analysis. The analysis of the compliance with the Hardy–Weinberg distribution for the population group, the χ^2^ analysis for the trend and the calculation of the OR were conducted with the assistance software at site: http://ihg.gsf.de/cgi-bin/hw/hwa1.pl. Unconditional logistic regression analysis was used for calculating OR and confidence interval (CI). The Bonferroni correction for multiple testing was not used. The remaining analyses were carried out using the Statistica 10.0 (StatSoft Inc.) program.

## Results

Demographic and clinical characteristics of the patients and controls covered by this study are presented in Table 1. Analysis of association of *IL1A* and *IL1B* gene polymorphisms in *H. pylori*-infected patients with chronic gastritis was performed separately for each gene.

Patients with chronic gastritis, chronic gastritis with atrophy, chronic gastritis with intestinal metaplasia and chronic gastritis with dysplasia were assigned to one group of patients with chronic gastritis. This yielded four groups for molecular analysis: population group, control group without lesions and without infection of *H. pylori* in gastric mucosa, chronic gastritis with *H. pylori* infections, and gastric cancer of intestinal type. Population group consisted of 100 healthy individuals (50 females, 50 males) especially collected for studies. Age distribution among population group was 21–30, in control group 16–57 and in patient group 11–87 (Table 1).

**Fig. 1:**
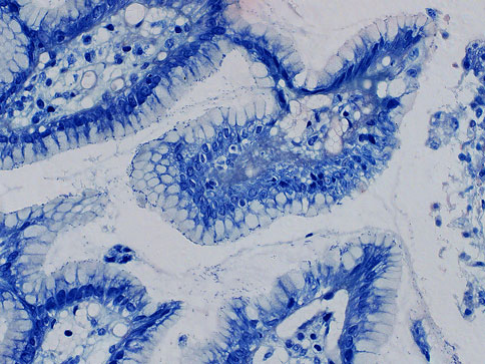
*Helicobacter pylori* by Giemsa-Romanovsky staining. *H. pylori* (medium stage of infection) present in a layer of mucus covering the gastric epithelium (magnification ×400)

**Fig. 2:**
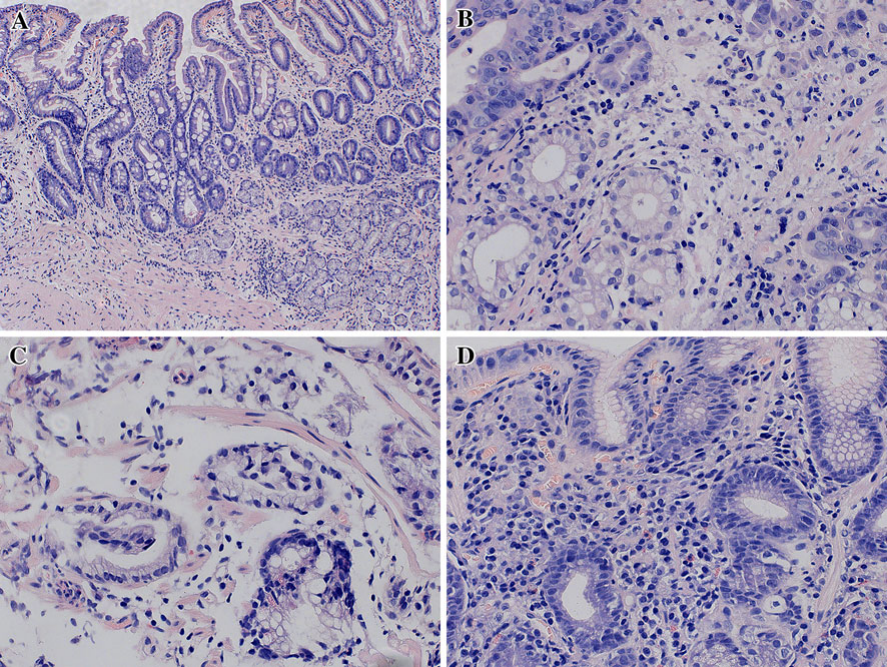
Hematoxylin and eosin staining of different stages of mucosa changes. **a** Moderate atrophy (grade 2) and glands with signs of intestinal metaplasia (grade 2) in pylorus (magnification ×100). **b** Gastric cancer (tubular adenocarcinoma G2-Kubo classification; intestinal type: Lauren classification). Solid nests, cancerous glands of adenocarcinoma (*upper*) and benign glands (*bottom left*). Cancer and inflammatory infiltration in the stroma (magnification ×400). **c** One gland with signs of severe stage dysplasia (grade 3; *right bottom*) and benign glands (*left* and *upper*) (magnification ×400). **d** Chronic gastritis (grade 2). Gastric mucosa with inflammatory infiltrate composed of lymphocyte cells, plasma cells and neutrophilic (magnification ×400)

Histological examinations were performed according to the updated Sydney system using four grades. Examples of biopsies specimens with different stages of gastritis and *H. pylori* infection are presented on Figs. 1 and 2.

The *IL1A* and *IL1B* genotype distribution, allele frequencies, common nomenclature, methodological nomenclature, methods used for genotyping are summarised in Table 2. Methodical nomenclature recommended by Human Genome Variation Society was used (www.hgvs.org). The *IL1A* and *IL1B* genotype distribution were in Hardy–Weinberg equilibrium.

**Table 2:** Polymorphisms in the *IL1A* and *IL1B* genes and methods of their genotyping

Gene	SNP number (db SNP)	Common nomenclature used in paper (alleles)	Methodological nomenclature	Genotyping method RFLP (bp)	Control group *n* = 13	Patients *n* = 131	Population group *n* = 100	Significance
Allele dose effect								
*IL1A*	rs1800587	Alleles *Nco*I	–889 °C > T					
		NN		98	CC = 8 (61.5 %)	CC = 60 (46 %)	CC = 48 (48 %)	Population + control vs. patients OR = 1.25; *p* = 0.24 95 % CI= [0.857–1.830] Population vs. patients OR = 1.21; *p* = 0.40 95 % CI = [0.809–1.770]
		Nn		98, 82, 16	CT = 4 (30.8 %)	CT = 49 (37 %)	CT = 41 (41 %)	
		nn		82, 16	TT = 1 (7.7 %)	TT = 22 (17 %)	TT = 11 (11 %)	
Allele frequency significance				Patients vs. population *p* = 0.1536		Patients vs. population + control *p* = 0.1172		
Effect of recessiveness and dominance								
*IL1A*	rs1800587	NN + Nn			CC + CT = 12 (92 %)	CC + CT = 109 (83 %)	CC + CT = 89 (89 %)	Population + control vs. patients OR = 1.16; *p* = 0.16 95 % CI = [0.799–3.610] Population vs. patients OR = 1.63; *p* = 0.21 95 % CI = [0. 751–3.549]
		nn			TT = 1 (8 %)	TT = 22 (17 %)	TT = 11 (11 %)	
*IL1A*	rs1800587	NN			CC = 8 (62 %)	CC = 60 (46 %)	CC = 48 (48 %)	Population + control vs. patients OR = 1.16; *p* = 0.56 95 % CI = [0.702–1.925] Population vs. patients OR = 1.09; *p* = 0.74 95 % CI = [0.648–1.840]
		Nn + nn			CT + TT = 5 (38 %)	CT + TT = 71 (54 %)	CT + TT = 52 (26 %)	
Allele dose effect								
*IL1B*	rs1143634	Alleles TaqI	+3954C > T					Population + control vs. patients OR = 1.54; *p* = 0.03 95 % CI = [1.030–2.304] Population vs. patients OR = **1.58;** *p* = **0.03** 95 % CI = [1.039–2.403]
		TT		126, 104	CC = 7 (54 %)	CC = 68 (52 %)	CC = 63 (63 %)	
		Tt		230, 126, 104	CT = 5 (38 %)	CT = 42 (32 %)	CT = 28 (28 %)	
		tt		230	TT = 1 (8 %)	TT = 21 (16 %)	TT = 9 (9 %)	
Allele frequency significance				Patients vs. population *p* < 0.0001		Patients vs. population + control *p* < 0.0001		
Effect of recessiveness and dominance								
*IL1B*	rs1143634	TT + Tt			CC + CT = 12 (92 %)	CC + CT = 110 (84 %)	CC + CT = 91 (91 %)	Population + control vs. patients OR = **1.97;** *p* = **0.09** 95 % CI = [0.884–4.375] Population vs. patients OR = 1.93; *p* = 0.16 95 % CI = [0.843–4.422]
		tt			TT = 1 (8 %)	TT = 21 (16 %)	TT = 9 (9 %)	
*IL1B*	rs1143634	TT			CC = 7 (54 %)	CC = 68 (52 %)	CC = 63 (63 %)	Population + control vs. patients OR = 1.51; *p* = 0.15 95 % CI = [0.904–2.516] Population vs. patients OR = **1.58;** *p* = **0.09** 95 % CI = [0.92**7**–2.684]
		Tt + tt			CT + TT = 6 (46 %)	CT + TT = 63 (48 %)	CT + TT = 37 (37 %)	

Methodological nomenclature recommended by Human Genome Variation Society (www.hgvs.org). Statistically significant values are indicated in bold

**Fig. 3:**
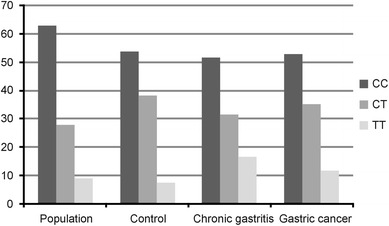
Comparison of genotype frequencies of +3954C>T polymorphism of *IL1B* gene among studied groups of patients with population and control group. Significantly different distribution was observed in comparison of chronic gastritis patients with population group. OR: 1.609; 95 % CI: 1.05–2.47; χ^2^ = 4.72; *p* = 0.03

 In the case of *IL1A* gene polymorphisms no significant differences in genotype distribution and allele frequencies between patients infected with *H. pylori* and chronic gastritis, chronic atrophic gastritis, intestinal metaplasia, dysplasia or gastric cancer and the control or population were observed, either in dominant or recessive model of inheritance. However, when the genotype distribution of +3954C>T polymorphism of *IL1B* gene was analysed in the same group of patients, control and population group, significant differences were found in the dominant model of inheritance. Frequency of allele T of +3954C>T polymorphism of *IL1B* gene was higher in group of patients with chronic gastritis, atrophy, intestinal metaplasia, dysplasia or intestinal type of gastric cancer (32.1 %) as compared with population group (23 %), χ^2^ = 4.61 and *p* = 0.03. This corresponds to OR: 1.58, 95 % CI: 1.04–2.4. Genotype distribution in all studied groups is presented in Fig. 3.

## Discussion

The present study examined the role of IL1A and IL1B polymorphisms and the co-existence of *H. pylori* infection in the susceptibility to the development of chronic gastritis in group of patients from Western Poland. For the first time, we analysed the *IL1A* and *IL1B* gene polymorphisms as potential factors predisposing, together with *H. pylori* infection, to pathological changes sharing the same localisation in the digestive tract.

The involvement of *H. pylori* in the development of chronic gastritis and cancer has already been confirmed and is widely accepted (IARC Working Group 1994). Interactions occurring between the bacterium and cells of the gastric mucosa contribute to the formation of an inflammatory infiltration made up of neutrophils, lymphocytes, plasma cells and macrophages. *H. pylori* stimulates directly intracellular signalling cascade leading to the activation of the nuclear factor-κB (Sobala et al. 1991), which in combination with the tumour necrosis factor (TNF)-α and IL-1 plays an important role in this process (Matsukura et al. 2003). The process can also be activated by the *H. pylori* infection of the gastric mucosa cells via the mitogen-activated protein kinase pathway (Hwang et al. 2002; Matsukura et al. 2003; Yamaoka et al. 1997). The increase in the activity of TNF-α correlates with the degree of progress of histological changes of the gastric mucosa inflammation linked with this infections (Hurme et al. 1998). It is capable to activate many genes associated with the host inflammation and immunological response including genes coding several dozen different cytokines, chemokines as well as receptors involved in the process of immunological recognition (di Giovine et al. 1992). Recently, researchers have been focusing their increasing attention towards the link of polymorphisms of definite human genes with the occurrence of diseases. Such link was demonstrated to exist, among others, for the gene *IL1* (Pociot et al. 1992). The presence of various proinflammatory IL-10 genotypes, TNF-α, IL1B and the IL-1 receptor antagonist increases the risk of development of gastric carcinoma. Such role of the proinflammatory IL-1 polymorphism (IL1RN*2, IL1B–511T/–31C) was demonstrated in experiments reported by El-Omar et al. (2000, 2001). However, this link was not confirmed by investigations carried out in Korea by Ryu et al. (2002) as well as in Japan by Kato et al. (2001). Similar conclusions can be drawn from experiments conducted on Taiwan by Wu et al. (2003). In addition, differences were demonstrated in the occurrence of the *IL1B* polymorphism within Asian populations. In the case of the Japanese population, IL1B–511C>C polymorphism was found dominant among patients with advanced atrophic chronic gastritis, whereas IL1B–511T>T+T>C polymorphism dominated among the Chinese population. No differences were determined in the frequency of occurrence of C and T alleles in Tai and Vietnamese populations (Matsukura et al. 2003). Very interesting conclusions can be drawn from the paper of Zeng et al. (2003) demonstrating that the IL1B–511T>T genotype exhibits association with gastric carcinoma in regions of China with both high and low incidence of gastric carcinoma. In the case of the IL1B–31C>C genotype, it occurred more frequently in patients with gastric carcinoma than in the control group in the region characterised by high gastric carcinoma incidence, whereas in the region with low gastric carcinoma incidence, it was found more frequent in the control group than in patients with cancer. This indicates that the T allele can act as a proinflammatory allele in genotype IL1B–31T and both genotypes can constitute independent gastric carcinoma risk factors. These suggestions were corroborated by the results of investigations carried out in Korea (Chang et al. 2005) indicating the significance of the *H. pylori* infection and the presence of IL1B–31T/IL1B–511C polymorphism for the increase by the gastric mucosa of IL1B production and, consequently, the development of the intestinal type of gastric carcinoma. In the case of the IL1B +3954T polymorphism, El-Omar et al. (2000) failed to demonstrate its proinflammatory impact on the gastric mucosa and even suggested its protective action against the development of gastric carcinoma. Also other researchers failed to demonstrate association of this polymorphism with risks of gastric carcinoma occurrence (Palli et al. 2005; Sicinschi et al. 2006; Zeng et al. 2003).

Among available meta-analyses very interesting is one performed by Wang et al. (2007), who collected data from 39 studies, which included 6,863 gastric cancer cases and 8,434 controls. The summary OR of gastric cancer risk associated with IL1B–511T, 231C, 13954T and IL1RN*2 was 1.26 (95 % CI: 1.03–1.55), 1.00 (95 % CI: 0.82–1.22), 1.37 (95 % CI: 0.94–2.00) and 1.20 (95 % CI: 1.01–1.41), respectively. IL1B–511T was associated with an increased risk of gastric cancer (intestinal type) (OR: 1.76, 95 % CI: 1.12–2.57). IL1RN*2 was also associated with an increased risk of gastric cancer among Caucasians (OR: 1.30, 95 % CI: 1.09–1.54).

Another meta-analysis based on fourteen studies on the *IL1B* +3954 polymorphism covering data from six Asian and eight non-Asian populations showed lack of statistical significance between presence of studied polymorphism and risk of gastric cancer. In general, all results were similar in magnitude when analyses were restricted to *H. pylori*-positive cases and controls (Persson et al. 2011). The results indicate the importance of stratification by anatomic site, histologic type, *H. pylori* infection and country of origin. Study quality considerations, both laboratory and epidemiologic, can also affect results and may explain the variability in results published to date.

In other studies of IL1B +3954 polymorphism weak dominant effect of putative-susceptible T allele is suggested. No significant associations were found regarding the IL1B +3954 polymorphisms associated with gastric cancer but the number of eligible studies on IL1B +3954 polymorphisms is rather limited and all findings should also be explained with extreme caution. In this meta-analysis IL1B −511T allele and IL1RN*2 variable number of tandem repeats (VNTR) are significantly associated with an increased risk of developing gastric carcinoma and even more significantly with non-cardia gastric carcinoma or with intestinal type gastric carcinoma. Both are significantly associated with an increased risk of developing gastric carcinoma among Caucasians, but not among Asians or Hispanics. IL1B–31C allele or homozygous CC plus TT, or IL1B +3954T allele, however, are not associated with an increased risk of developing gastric cancer, but IL1B–31 homozygous CC plus TT is significantly inversely associated with the risk of intestinal type gastric cancer (Xue et al. 2010).

Meta-analysis performed by Camargo et al. (2006) covered the presence of *IL1B*–*511T, IL1B*–*31C, IL1B* +*3954T*, or IL1RN*2 alleles and their association with gastric cancer risk. For IL1B +3954T polymorphisms eight studies were cited: four in Caucasians, three in Asians, and one in Hispanics. Individuals carrying the T allele had a nonsignificantly elevated gastric cancer risk compared with the C/C genotype. Subgroup analyses by histologic subtype and location were not done in any of the ethnic groups due to insufficient data for this SNP. Another type of limitation was the small number of studies and, consequently, limited statistical power. Because gastric cancer is a multifactorial disease, more studies should focus on testing haplotypes and gene–environment interactions like *H. pylori* infection, as this might elucidate further the genetics of this complex disease (Camargo et al. 2006).

On the other hand, our investigations carried out for Polish general population concerning the analysis of the association of the *IL1B* gene +3954C>T polymorphism with inflammatory changes of the gastric mucosa and with the intestinal type of gastric carcinoma in patients with coexisting *H. pylori* infection demonstrated such a link. Therefore, the occurrence of this polymorphism, similarly to the IL1B–31/–511 polymorphism, may be treated as an additional risk factor in the development of gastric mucosa inflammation and carcinogenesis. Moreover, the existence of other synergistically acting polymorphisms should also be taken into consideration. This conjecture appears to be corroborated by reports of Korean researchers (Lee et al. 2004). They demonstrated that in IL1B–1473G carriers, the risk of the intestinal type gastric carcinoma increases.

Gene *IL1A* possesses three polymorphisms: in –889 and 4845 positions as well as at 46 bp VNTR in intron 6 (Dinarello 1996). According to recently published investigations carried out on Korean patients suffering from gastric carcinoma, no association of the occurrence of IL1A–889 polymorphism with the risk of development of gastric carcinoma was demonstrated (Chang et al. 2005). In our studies, we assessed the association of IL1A polymorphism in –889 position with the risk of occurrence of inflammatory changes of the gastric mucosa and of the intestinal type gastric carcinoma in patients infected with *H. pylori*. It showed only an increasing tendency of the frequency of the occurrence of C and T alleles as well as of the polymorphisms genotype –889C>T of the *IL1A* gene with no statistical significance in the examined group of patients.

Our research results corroborate a significant role of the occurrence of gene *IL1* polymorphisms in the development of the inflammation of gastric mucosa as well as in the gastric carcinoma development. It is also necessary to take into account the significance of gene polymorphisms of other cytokines (e.g. IL-6, IL-10, TNF-α) as well as other genetic factors which can exhibit a synergistic action in the development of these changes. The determination of the risk level of gastric carcinoma development must take into consideration many environmental factors such as, among others, salt consumption or smoking. Another very important factor is the scale of occurrence, in a given population, of the *H. pylori* infection. Differences in the occurrence of individual environmental factors can exert a significant influence on the research results conducted in a specific population. That is why attempts at improving the determination accuracy of gastric carcinoma risks in a given population should include a greater number of the examined polymorphisms and take into account broadly understood environmental factors as well as ethnic and geographical differences.
